# Proposal of a New Therapeutic Classification in Gingival Smiles Focused on Treatment with Semi-Permanent Infiltrations

**DOI:** 10.3390/dj12100319

**Published:** 2024-10-05

**Authors:** Gema Angulo-Manzaneque, María Baus-Domínguez, Gonzalo Ruiz-de-León, María-Ángeles Serrera-Figallo, Fátima S. Aguilera, Daniel Torres-Lagares

**Affiliations:** 1 Department of Stomatology, Faculty of Dentistry, University of Seville, C/Avicena S/N, 41009 Seville, Spain; ruizdeleong@gmail.com (G.R.-d.-L.); maserrera@us.es (M.-Á.S.-F.); danieltl@us.es (D.T.-L.); 2 Department of Stomatology, Faculty of Dentistry, University of Granada, Colegio Máximo, Campus Universitario de Cartuja, 18071 Granada, Spain; fatimas@ugr.es

**Keywords:** gummy smile, treatment, classification, muscle hypertonicity, hyaluronic acid, gingival smile, smile esthetics

## Abstract

A gummy smile, defined as excessive gingival exposure while smiling, is an esthetic and functional condition affecting an individual’s quality of life. Despite its prevalence and impact, the classification and treatment of the gummy smile remain challenging in clinical practice. The problem lies in (1) the fact that the etiology of this pathology is multifactorial, and these factors sometimes go unnoticed, (2) the lack of consensus on the classification criteria, which, together, create (3) challenges in designing an optimal treatment plan for each patient. This article reviews the etiologic factors of this condition as the main basis for understanding the existing classifications of the gummy smile. It highlights the importance of muscle dynamics in the genesis and treatment of this clinical condition. We present a new, treatment-oriented classification that integrates the muscle hyperactivity present within the classification criteria and explore the implication of this interaction in the design of effective treatments. The ultimate goal of this present work is to improve the clinical understanding of the gingival smile and offer more personalized treatment strategies, through a more complete classification.

## 1. Introduction

A Gummy smile, characterized by excessive gingival exposure while smiling, is a multifactorial phenomenon involving dental, skeletal, and muscular aspects. According to Mazzuco et al. [1], a gummy smile is defined as the display of more than 3 mm of gingiva while smiling. This condition can affect both facial esthetics and oral function, making it an area of significant interest in contemporary dentistry.

Despite its prevalence and clinical relevance, the classification and treatment of gummy smiles remain challenging in clinical practice. The lack of consensus on classification criteria hinders the accurate identification of the underlying etiology, which, in turn, complicates the planning of effective treatment within the various therapeutic options that exist. In addition, the differences in treatment proposals reflect the diversity and discrepancy in understanding this condition [2,3].

This paper aims to provide a comprehensive view of the etiological factors and existing classifications of the gummy smile and explore the relationship between muscle hyperactivity, bone and fat volume deficit, and this phenomenon. Its aim is to propose a new classification with a particular focus on how the facial muscles and lack of bone and fat structure contribute to the gummy smile, leading to correct treatment proposals.

This study was conducted using a scoping review methodology to map and analyze the existing literature on the classification and treatment of the gummy smile, focusing on interventions with semi-permanent infiltrations. The literature search was carried out in databases such as PubMed and Google Scholar, covering publications from 1973 to 2023 in English and Spanish. Keyword combinations such as “gummy smile”, “gingival smile”, “botulinum toxin”, “Treatment”, “Classification”, “Muscle hypertonicity”, and “smile aesthetics” were used, applying Boolean operators to maximize the relevance of the results.

Studies that presented classifications and treatments of the gingival smile and those that analyzed the use of botulinum toxin and other semi-permanent infiltrations, such as autologous fat, were included [4,5]. Articles that did not focus on this topic and those that did not provide original data were excluded. The selected studies were organized thematically, allowing the identification of key trends and gaps in the current literature, which are discussed to propose a new therapeutic classification.

The ultimate goals of this article are to improve our understanding of the gummy smile and provide more precise guidelines for its classification and treatment. This will allow for more effective and personalized care for patients affected by this esthetically and functionally challenging condition.

## 2. Etiology

One of the major etiologic factors contributing to gummy smiles is the anatomic position of the teeth and jaws. Individuals with a short upper lip, a high-positioned jaw, or a tendency to make gummy smiles are more likely to exhibit excessive gingival display [6,7,8]. Additionally, certain orthodontic conditions, such as deep or vertical maxillary overbite, can cause a gummy smile [2,9].

Another important etiologic factor is the level of gingival inflammation and the degree of hyperplasia of the gingival tissue. Gingivitis, a common inflammatory condition of the gums, can lead to gingival enlargement and contribute to the appearance of a gummy smile. Factors that influence the development of gingivitis, such as poor oral hygiene, plaque accumulation, and certain systemic conditions such as diabetes, may also play an incidental role in the etiology of the gummy smile [3].

Following the multifactorial etiology of the gummy smile, muscle hyperactivity is added to these problems. Mimetic facial muscles (MFM), such as the levator labii superioris and ala nasalis (LLSAN) and levator labii superioris (LLS), are crucial in smile dynamics, although their interaction is not yet fully understood [10,11].

Factors contributing to the gummy smile include alterations in skeletal development, altered passive eruption of the teeth, hyperactivity of the levator labii superioris alaeque nasi muscles, and upper lip anatomy. Specifically, hyperactivity of levator muscles, such as the levator labii superioris alaeque nasi, can cause excessive lip elevation during smiling, exposing more gingiva than normal [12,13].

According to Pavone et al. [14], anatomical variations and mobility of the upper lip are determining factors in the classification and management of this condition. From an orthodontic perspective, a gummy smile may also be related to skeletal discrepancies, such as an excessively long upper jaw or maxillary protrusion, contributing to gingival exposure [14,15]. Furthermore, according to a systematic review by Londono and Botero [16], esthetic preferences vary, but a smile with minimal gingival exposure is generally considered more attractive.

Treatment of the gingival smile may include a variety of interventions, ranging from surgical approaches, such as gingivectomy and orthognathic surgery, to less invasive treatments, such as botulinum toxin application to reduce muscle activity and thus gingival exposure during smiling [1,10]. The choice of treatment depends on the underlying etiology and the patient’s esthetic expectations, as highlighted by the studies of De Maio [6] and Patel et al. [10].

Understanding facial muscles’ interactions and functions in the gingival smile is essential for effective diagnosis and treatment. This requires a detailed evaluation of the facial anatomy, muscle dynamics, and esthetic interactions [15,17,18].

According to the pulley and lever system explained by Di Maio [14,16], the lack of bony and fatty support in the lower third causes an imbalance in the ability of the depressor muscles to counteract the action of the levator muscles during the muscular movement of the face. When smiling, this results in increased force or hypermobility of the elevators, as they are not adequately counteracted.

**Figure 1:**
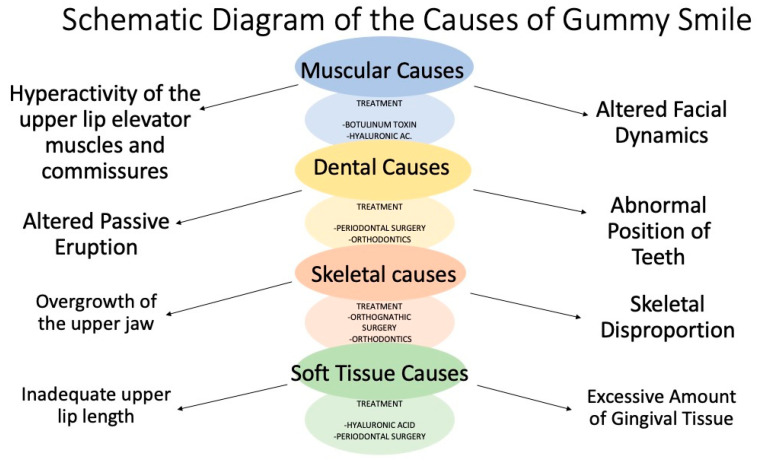
Muscular causes: pastel blue color identifies factors related to facial musculature that affect gingival exposure; lighter blue color identifies treatment. Dental causes: pastel yellow color identifies causes related to structure and position of teeth; lighter yellow color identifies treatment. Skeletal causes: soft orange color highlights bony aspects that influence appearance of smile; lighter orange color indicates treatment. Soft tissue causes: pastel green color addresses soft tissue factors such as lips and gingiva; lighter green color indicates treatment.

In our opinion, only through this personalized approach can successful treatment strategies be designed that address both the esthetic and functional aspects of the gingival smile. Hence, the role played by the various classifications and the therapeutic approach to them is an important, if not critical, point (Figure 1). This diagram is useful to comprehensively visualize how different biological and structural factors can contribute to the gingival smile, facilitating the understanding of this phenomenon in a clinical and academic context and providing a correct treatment [13,19,20,21].

## 3. Classifications

Several attempts have been made to classify the gingival smile (Table 1) [1,12,22,23,24,25,26,27,28,29,30]. Some investigators have classified gingival smiles based on the underlying etiology, such as skeletal, dentoalveolar, or muscular factors. This approach can help identify the root cause of the gingival presentation and guide appropriate treatment strategies (depending on which area is to be treated).

Other classifications have focused on the degree of gingival exposure, classifying gingival smiles as mild, moderate, or severe based on the percentage of gingival tissue exposed during a smile. This type of classification can help assess the condition’s esthetic impact and determine the appropriate therapeutic approach (based on the improvement that can be expected from therapeutic intervention).

Although there is no single universally accepted classification system for gingival smiles, existing frameworks provide valuable information on the various factors contributing to this condition and appropriate clinical management strategies, although they still have significant limitations.

As a prime example of these limitations, and after reviewing attempts to classify and study the gummy smile over 50 years, both through literature research and clinical observations, we can observe that, even when treating the gummy smile by interventions on the levator and zygomatic muscles for muscle hypermobility with botulinum toxin or myomodulation and volume replacement with fillers [14,16], in most cases these measures prove insufficient [31,32,33].

In recent years, the use of semi-permanent infiltrations with hyaluronic acid has significantly increased in demand and application in the field of orofacial harmonization, including for the treatment of the gummy smile [34].

In our opinion, an additional subclassification, with a focus on the lip and its dynamics, is needed, specially designed for the treatment of the gingival smile with semi-permanent infiltration. This aspect has led us to propose the following classification, which can and should complement the existing ones.

## 4. Proposed Classification of the Gingival Smile with a Focus on Semi-Permanent Infiltration Treatment

The proposed classification is based on a thorough evaluation of the facial anatomy, including the relationships between teeth, bone structure, and surrounding soft tissues. In addition, considering the great importance of the function of the facial muscles involved in the smile, we focus primarily on muscular hypermobility and the lack of bony and fatty support of the jaws.

The proposed classification is based on identifying the different alterations present in the patient, corresponding to a specific therapeutic approach by infiltrating semi-permanent fillers.

Proposed subclassification “Gingival Balance Face”:

- Type 1.—Upper lip vermilion red refinement.
- Type 2.—Loss or lack of bone and fat volume.
  a. Upper jaw.
  b. Piriform fossa.
  c. Chin.
- Type 3.—Muscle hypermobility.
  a. Lift muscle of the upper lip and wing of the nose (LLSAN).
  b. Levator labii superioris muscle (LLS).
  c. Nasal septal depressor muscle (myrtiformis).
  d. Zygomaticus major (ZM) and zygomaticus minor (Zm) muscles.

Type 1.—Upper lip vermilion red refinement

**Figure 2:**
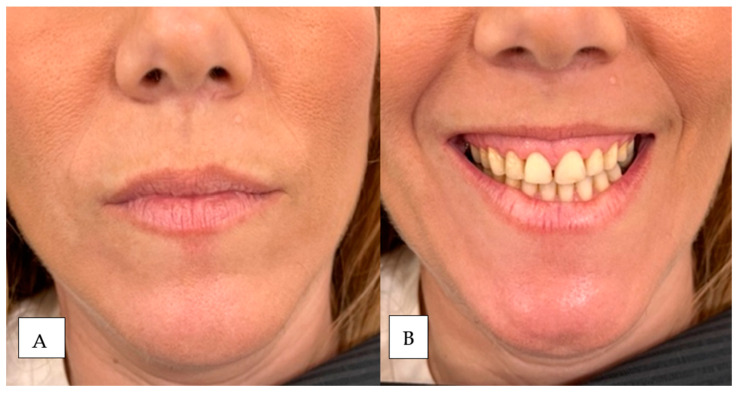
In image (**A**) (left) the patient is at rest and in image (**B**) (right) maximum smile. The patient has moderate upper lip volume at rest and is sagging in the piriform fossa. After smiling, the red of the vermilion disappears due to the lip’s inversion and the nasolabial folds’ marking.

**Figure 3:**
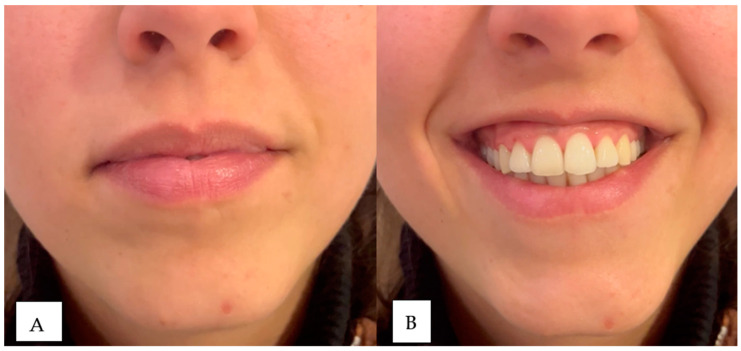
In image (**A**) (left) the patient is at rest and in image (**B**) (right) in maximum smile. The patient has a moderate upper lip volume at rest that disappears after smiling due to the reversal of scarlet red.

This subcategory addresses the inversion and excessive strength of the orbicularis oris muscle in the smile, which contributes to the thinning of the lip tissue and increased gum exposure when smiling. For its diagnosis, we ask the patient to smile, and if we observe that the only movement of the smile that exposes gum is because the upper lip is inverted inward, we would have this classification (Figure 2; Figure 3).

Type 2.—Loss or lack of bone and fat volume

**Figure 4:**
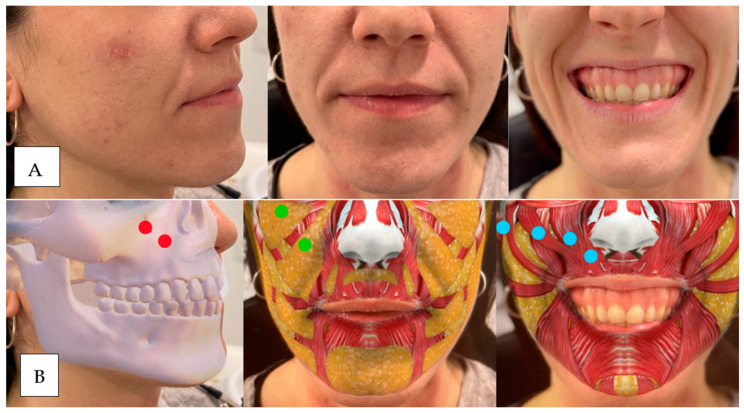
(**A**) The patient has lost maxillary and piriform fossa support, which causes sinking and marking of the nasolabial folds in static and dynamic positions. Muscular hypermobility of the levator and zygomatic muscles and inversion of the upper lip are observed. (**B**) Injection points. Red: upper maxilla above and piriform fossa below. Green: upper medial maxillary and lower nasolabial fat. Blue: right to left: zygomaticus major, zygomaticus minor, levator labii superior, levator labii superior, and ala of the nose. Note: the tool used to generate the graphic overlays of muscles, bone structures, or adipose tissue was an Instagram filter developed by Praxis Studio^®^, The Anatomy Lab (Cúcuta, Col.).

**Figure 5:**
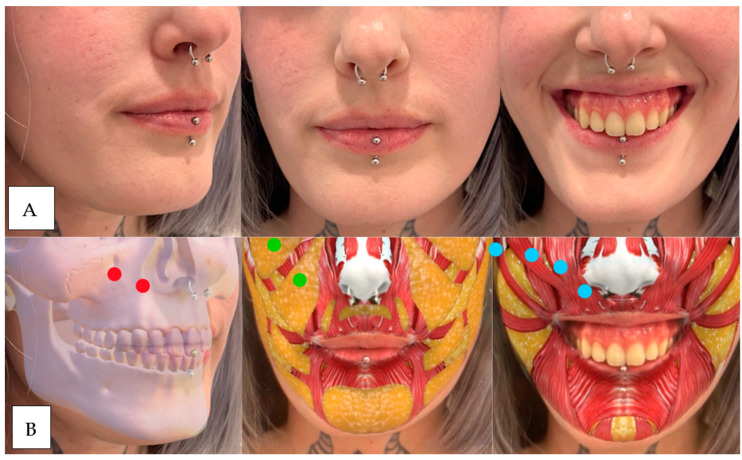
(**A**) We observe sagging in the maxillary region and piriform fossa due to a lack of bone and fat support. At the muscular level, we can see hypermobility of the levator and zygomatic muscles without marking the nasolabial fold in static. The upper lip does not show inversion. (**B**) Red: upper maxilla above and piriform fossa below. Green: upper medial maxillary and lower nasolabial fat. Blue: right to left: zygomaticus major, zygomaticus minor, levator labii superior, levator labii superior, and ala of the nose.

**Figure 6:**
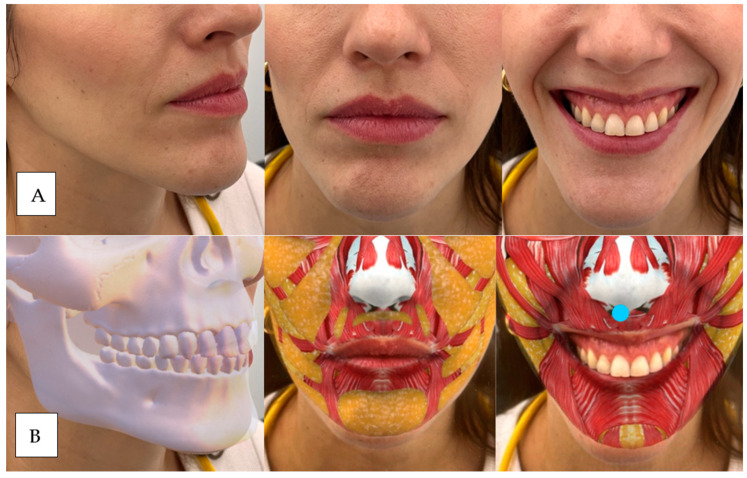
(**A**) The patient presents zygomatic muscle hypermobility as she elevates the commissures, with thinning of the red of the vermilion and drooping of the tip of the nose (depression of the nasal septum, blue dot). (**B**) The blue dot indicates where we would do the nasal septum treatment.

**Figure 7:**
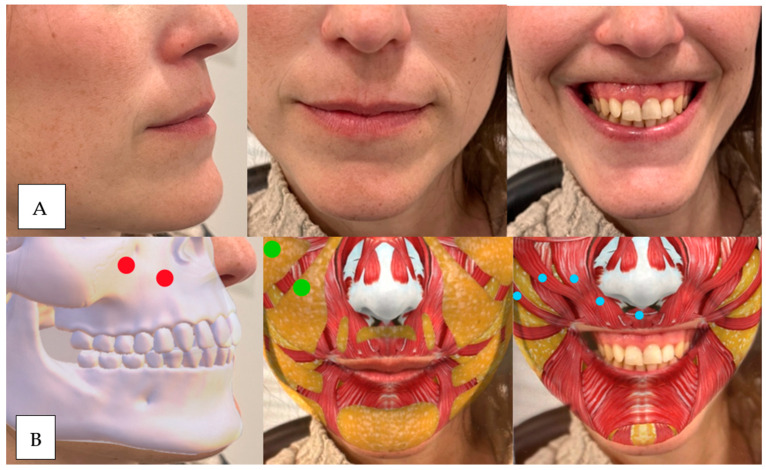
(**A**) The patient presents subsidence in the piriform fossa, continuous hypermobility of the levator and zygomatic muscles, subtle marking of the nasolabial fold, complete disappearance of the vermilion, and drooping of the tip of the nose (depression of the nasal septum) (**B**) Red: upper maxilla above and piriform fossa below. Green: upper medial maxillary and lower nasolabial fat. Blue: right to left: zygomaticus major, zygomaticus minor, upper lip elevator, upper lip elevator and ala of the nose, and nasal septum.

Patients who have some loss or lack of volume in the following regions tend to have gummy smiles due to a lack of blocking and counterbalancing (Figure 4, Figure 5, Figure 6, Figure 7).

a. Upper jaw: lack of bony projection and fat volume of the medial and nasolabial compartment.
b. Piriform fossa: lack of bony projection in the canine fossa.
c. Chin: Lack of bony projection and fatty volume of the mentonian compartment.

Type 3.—Muscle hypermobility

**Figure 8:**
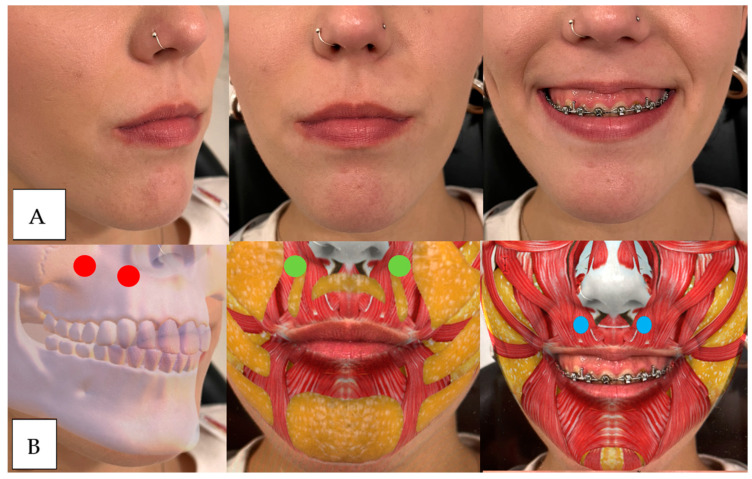
(**A**) From left to right unsmiling patients to maximum smile. We observe sinking in the piriform fossa and horizontal elevation of the upper lip, with a slight tendency towards medial by the LLSAN. (**B**) Red dot: upper maxilla above, piriform fossa below. Green dot: nasolabial fat. Blue dot: levator labii superioris muscle.

**Figure 9:**
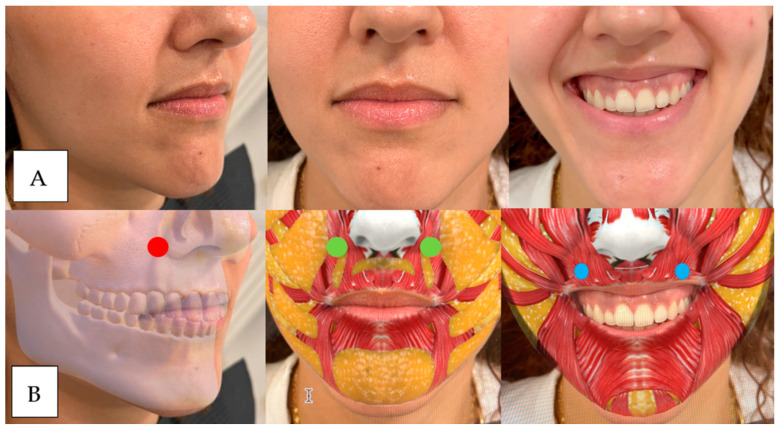
(**A**) From left to right, unsmiling patient to maximum smile. We observe sinking in the piriform fossa and in the smile, refinement of the vermilion at the level of the LLS insertion. (**B**) Red Dot: Up piriform fossa, Green dot: subnasal fat. Blue Dot LLS.

**Figure 10:**
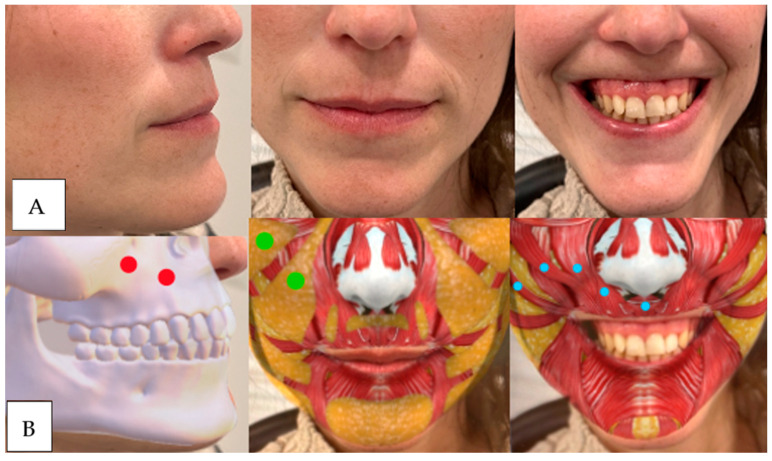
(**A**) From left to right unsmiling patient to maximum smile. We observe sinking in the piriform fossa, lack of maxillary support, complete eversion of the vermilion and drooping of the tip of the nose. (**B**) Red dot: above, upper maxilla and piriform fossa, green dot: above, ma-xillary fat, medial and below, nasolabial. Blue Dot: left to right: ZM, Zm, LLS, LLSAN in equilibrium and nasal depressor.

**Figure 11:**
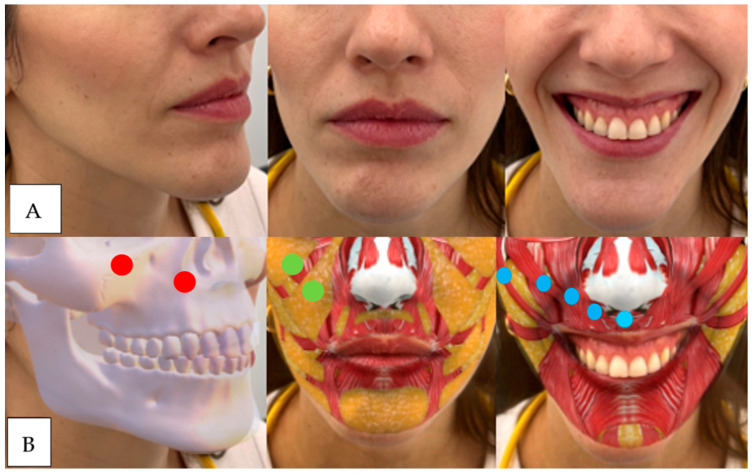
(**A**) From left to right unsmiling patients to maximum smile. We observe how the commissures rise upwards due to the hyperactivity of the zygomatics and how the nasal tip falls down. (**B**) Red Dot: Top, upper jaw and piriform fossa. Green dot: above, maxillary fat. Blue Dot: left to right: ZM, Zm, LLS, LLSAN and nasal depressor.

As we have already seen, hypermobility can be conditioned by a mismatch of forces and the lack of bone and fat support [14,16] (Figure 8, Figure 9, Figure 10, Figure 11).

a. LLSAN: this subcategory focuses on the action of the levator labii superioris and ala nasalis (LLSAN) muscles in the excessive elevation of the lips during smiling, which can result in increased gum exposure.
b. Levator labii superioris (LLS) muscle: similar to above, but also involves the levator labii superioris (LLS).
c. Nasal septal depressor muscle (myrtiformis): here, we consider the influence of the nasal septum or myrtiformis muscle and the levator muscles of the upper lip and ala of the nose, together with the orbicularis on the mobility of the lips, tip of the nose, and gingival exposure.
d. Zygomaticus major (ZM) and zygomaticus minor (Zm) muscles: we explored how traction of the zygomaticus muscles can contribute to a wide gingival smile by exposing the premolars, including the molars.

## 5. Application of the Proposed Classification to the Therapeutic Field

Clinicians can develop more accurate and personalized treatment plans by better understanding the various causes and mechanisms contributing to the gummy smile (Table 2).

It should be noted that hyaluronic acid treatment is generally safe and well tolerated, although it can present adverse effects and complications. Common effects include swelling, erythema, and bruising, which are temporary. Moderate side effects, such as nodule formation or infection, may also occur. More serious complications, although rare, include allergic reactions, tissue necrosis, and ischemic blindness. It is essential that practitioners carefully evaluate patients and discuss the risks and benefits of treatment to minimize these potential adverse effects [35,36,37,38,39].

In our therapeutic approach, which integrates the evaluation of muscle hypermobility and lack of bone and fat volume, we focus on volume replacement and myomodulation with hyaluronic acid as the main tools to address the esthetic and functional alterations associated with the gummy smile.

In relation to the treatment of a thinning of the upper lip vermilion, we will use an eversion technique involving microinjections of hyaluronic acid to provide volume and length to the vermilion’s red, thus restoring the harmony and proportion of the smile.

For treatment of the nasal septum, we observed that the tip of the nose tends to droop during smiling due to a lack of support. Therefore, we propose applying hyaluronic acid to the nasal spine and columella with a linear retroaction technique to provide structural support and block the action of the orbicular muscle.

For the treatment of the levator labii superioris and ala nasalis muscle (LLSAN), we will reposition the lost volumes in the piriformis and maxillary fossa, reaching the periosteum with 27 G water and a cross-linked hyaluronic acid, to provide support and volume. We will use microinjections of hyaluronic acid in the deep dermis with a 25 G cannula in retro tracer and fan to give weight and produce myomodulation, thus restoring the function and esthetics of the smile.

In treating zygomatic muscles and chin retraction, we will address chin retraction and volume loss in the periosteum with microinjections of hyaluronic acid. In more severe cases, we will use cannulation to ensure even product distribution throughout the chin adipose area to restore facial architecture effectively.

**Figure 12:**
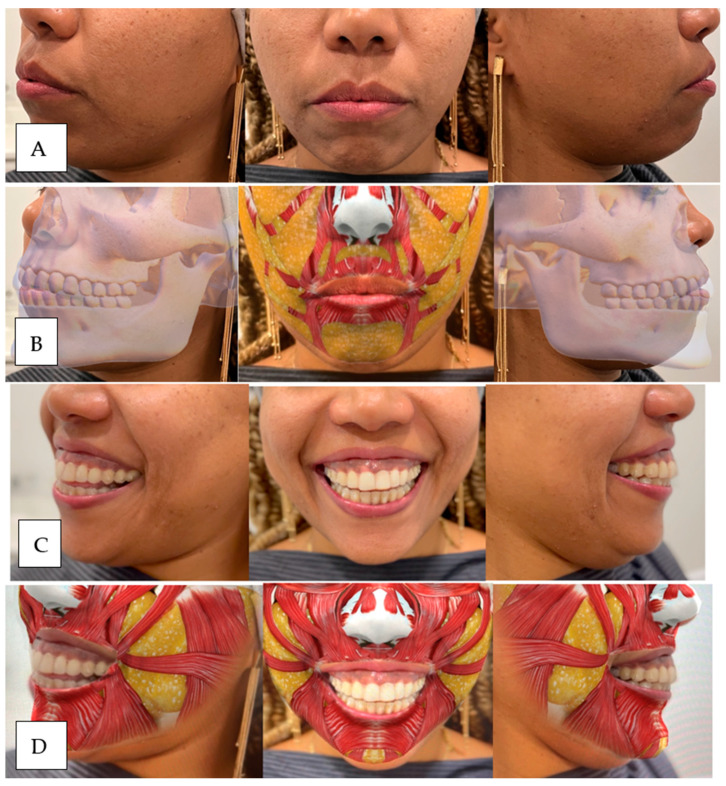
(**A**) Patient with lack of bone and fat volume in upper jaw, piriform fossa, and chin, without smiling, in which we observe some hypertonicity to create a lip seal and retrognathia in profile. (**B**) Anatomical diagram of the patient with lack of bone and fat volume in the upper jaw, piriform fossa, and chin, without smiling. (**C**) Patient with lack of bone and fat volume in upper jaw, piriform fossa, and chin, smiling; we observe that the upper lip has medium volume at rest that after smiling is inverted, becoming red vermilion with marking of nasogenian furrows. (**D**) Anatomical diagram of the patient with lack of bone and fat volume in the upper jaw, piriform fossa, and chin, smiling.

**Figure 13:**
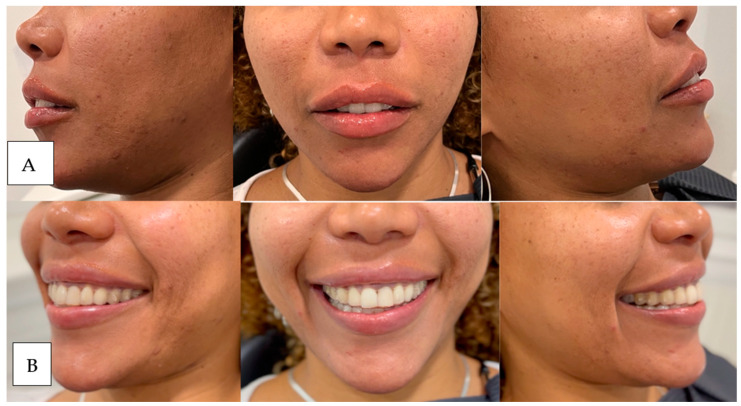
(**A**) After completing the gummy smile treatment with volume compensation and myomodulation with hyaluronic acid, we used 11 mL for bone support and fat volume replacement with cross-linked hyaluronic acid deposits in the periosteum. (**B**) Patient smiling after treatment myomodulation of the levator and zygomatic muscles with retroaction technique with a cannula in the deep dermis, ending with lip eversion.

In summary, our comprehensive therapeutic approach combines volume replacement and hyaluronic acid myomodulation to address the various causes of gummy smiles, providing optimal esthetic and functional results for our patients (Figure 12; Figure 13).

## 6. Conclusions

Our work presents a new classification that accompanies and complements the previous classifications and integrates the use of hyaluronic acid in the treatment of the gingival smile based on an exhaustive evaluation of muscular dynamics.

It is essential to point out that new studies are still needed to specifically address the role of the orbicular muscle and its strength when smiling, especially in cases where this strength causes the upper lip to invert, leaving it thin and with almost no scarlet red. The deficit of support and volume in the maxilla, piriform fossa, and chin and the imbalance that this presents in muscle mobility have also not been studied.

To improve the diagnosis and analysis of the gingival smile, the use of advanced technologies such as functional magnetic resonance imaging (fMRI), high-resolution 3D facial scanning, cone beam computed tomography (CBCT), and advanced electromyography can be very useful. These tools have the potential to provide a more accurate study of muscle activity and facial structure, which could optimize esthetic and functional outcomes in the treatment of the gingival smile. It is suggested that these technologies be explored in future studies [20,40,41,42].

Considering the inherent limitations of the proposed classification, it is essential to note that this initial framework may need to be adjusted as more clinical evidence is obtained. Therefore, we recommend that future research focuses on validating this classification in different populations and exploring the long-term impact of treatments with semi-permanent infiltrations. This will allow us to refine the classification and improve its clinical applicability, ensuring that it offers effective esthetic and functional solutions for a broader spectrum of gummy smile patients.

Understanding the dynamics of the facial muscles during smiling, including the role of the orbicular muscle, could provide a complete perspective on the etiology and management of the gingival smile, allowing more accurate and practical approaches to gingival smile treatment to be developed.

## Figures and Tables

**Table 1:** Classifications of the gingival smile are organized according to the approach applied by each author.

Classification Group	Authors	Factors Studied	Proposed Treatment	Main Conclusions
Muscular and Dento-Gingival Anatomy	Mazzuco [1], Mercado and Rosso [22], Monaco [23], Patel [24], Hao Wu [25]	Area and muscles of gingival exposure (Mazzuco): analyzes the amount and location of visible gingiva during the smile, identifying specific muscles such as the levator labii superioris and nasolabial (LLSAN) that contribute to different types of gingival smile. Esthetics, etiopathogenesis, and muscle functionality (Mercado and Rosso): they evaluate how smile appearance affects esthetic perception and underlying causes such as dental position and facial muscle activity. Dento-gingival and structural relationships (Monaco): this section considers altered passive eruption, upper lip length, and facial typology, which influence the smile’s appearance. Muscle mobility and control of gingival exposure (Patel): This section focuses on excessive muscle contraction and its impact on tooth and gingival exposure. It examines muscle mobility to classify the gingival smile. Detailed craniofacial variables (Hao Wu): uses precise measurements such as ANB, Pog-NP, and other facial dimensions to classify and better understand the craniofacial characteristics associated with different gingival smiles.	Botulinum Toxin: applied to reduce the hyperactivity of specific muscles involved in the gummy smile. Hyaluronic Acid: improves the support and projection of the maxilla and balance the muscular forces. Surgery and Orthodontics: in cases where the dentofacial structure requires it, orthognathic surgeries or orthodontic treatments can correct misalignments or structural problems.	Improved classification and treatment of the gingival smile based on specific anatomy and muscle functionality.
Esthetics and Facial Function	Garber and Salama [26], Saber and Ackerman [27], London and Botero [12]	Severity of gingival exposure, incisal-labial relationship (Garber and Salama): measures the amount of visible gingiva and the relationship between the incisal edge of the teeth and the upper lip at rest to classify the severity of gingival exposure. Facial harmony and lip movement (Saber and Ackerman): analyze how the lips move during smiling and their interaction with the teeth to determine the impact on overall facial esthetics. Physiological, anatomical, and functional characteristics (London and Botero): they evaluate facial symmetry, lips, gingiva, teeth, and smile dimensions.	Orthognathic and Periodontal Surgery: a more radical modification of the facial or gingival structure is needed for severe cases. Orthodontics and prosthodontics: less invasive treatments that seek to improve facial harmony and muscular functionality, including using orthodontic devices and dental esthetic techniques.	Emphasis on the importance of esthetics and facial functionality in treating the gingival smile, with proposals for personalized intervention.
Complex Customized Evaluations	Pavone [28], Tatakis [29], Oliveira [30]	Dental and periodontal analysis, individual characteristics (Pavone, Tatakis): this includes a detailed examination of the three-dimensional position of the teeth and clinical attachment levels. Subjective and objective evaluations of the smile (Tomas Oliveira): combines personal perception of the patient with objective measurements such as the shape and position of the teeth. Etiology of gingival excess and lip characteristics (Tatakis): focus on upper lip mobility and altered passive eruption.	Orthodontics and surgery for specific conditions. Personalized planning and multidisciplinary treatments to address specific causes identified in the evaluations.	A detailed and personalized evaluation of each patient leads to more effective and targeted treatments, recognizing the diversity of causes of the gummy smile.

**Table 2:** Application of the proposed classification to the therapeutic field.

Classification	Subcategory	Description	Evaluation Criteria	Treatment
1. Upper Lip Vermilion Red Refinement		To evaluate lip tissue thinning and orbicularis muscle inversion during smiling.	- Objective measurement of gingival exposure by photographic and video analysis. - Clinical evaluation of vermilion red thickness.	- Application of hyaluronic acid using standardized microinjection techniques to restore the volume and proportion of vermilion red. - Continuous evaluation of results through photographic and clinical analysis.
2. Bone and Fat Volume	2.1. Upper jaw	Identify loss or lack of volume in key areas.	Evaluation of bone projection and fat volume by 3D imaging and volumetric analysis. Patients usually have a flat midcheek.	- Bone and fat volume replacement using hyaluronic acid in specific areas (maxilla, piriform fossa, chin) both in periosteum with a 27 G needle and with a 25 G cannula in deep dermis. - Use of 3D imaging such as MRI and ultrasound to guide treatment delivery and adjustment.
	2.2. Piriform fossa		Analysis of the bony projection with computed tomography and clinical measurements. With the naked eye we can observe a subsidence or shadow in the area.	- Bone and fat volume replacement using hyaluronic acid in specific areas (maxilla, piriform fossa, chin) both in periosteum with a 27 G needle and with a 25 G cannula in deep dermis. - Use of 3D imaging such as MRI and ultrasound to guide treatment delivery and adjustment.
	2.3. Chi		Evaluation of fat and bone volume by MRI and clinical analysis. We will analyze with the Ricketts plane.	- Bone and fat volume replacement using hyaluronic acid in specific areas (maxilla, piriform fossa, chin) both in periosteum with a 27 G needle and with a 25 G cannula in deep dermis. - Use of 3D imaging such as MRI and ultrasound to guide treatment delivery and adjustment.
3. Muscle Hypermobility and Muscle Function.	3.1. Muscles elevating muscles of the upper lip and wing of the nose (LLSAN)	Analyze the excessive elevation of the upper lip.	-Electromyography evaluation. - Analysis of muscle dynamics during smiling.	- Myomodulation with hyaluronic acid and injection techniques in the affected areas. - Assessment of muscle response by electromyography and adjustments as needed.
	3.2. Levator labii superioris muscle (LLS)	To evaluate the contribution of the upper lip elevator to gingival exposure.	- Clinical examination. - Electromyography to measure muscle activity.	- Targeted myomodulation through injections to control upper lip levator muscle activity, adjusting gingival exposure.
	3.3. Nasal septal depressor muscle (myrtiformis).	To analyze the influence of the nasal septal depressor muscle on gingival exposure.	- Clinical evaluation. - Analysis of the dynamics during smiling.	- Myomodulation treatment to adjust nasal septal depressor muscle activity, evaluating results and modifying as needed.
	3.4. Zygomaticus major (ZM) and zygomaticus minor (Zm) muscles	To examine how zygomatic muscle traction contributes to the gingival smile.	- Measurement of muscle activity. - Analysis of facial dynamics.	- Comprehensive management of the muscular activity of the zygomatic muscles using myomodulation techniques and possible surgical interventions if necessary to optimize the gingival smile.

## Data Availability

The original contributions presented in this study are included in the article; further inquiries can be directed to the corresponding author/s.
